# A semi-structured interview for the dimensional assessment of internalizing and externalizing symptoms in children and adolescents: Interview Version of the Symptoms and Functioning Severity Scale (SFSS-I)

**DOI:** 10.1186/s13034-024-00788-y

**Published:** 2024-08-24

**Authors:** Jana Rausch, Leonard Bickman, Nina Geldermann, Felix Oswald, Danny Gehlen, Anja Görtz-Dorten, Manfred Döpfner, Christopher Hautmann

**Affiliations:** 1 School for Child and Adolescent Psychotherapy (AKiP), Faculty of Medicine and University Hospital Cologne, University of Cologne, Cologne, Germany; 2 Department of Psychology, Florida International University, Miami, FL USA; 3 Ontrak Health, Inc., Henderson, NV USA; 4 Department of Child and Adolescent Psychiatry, Psychosomatics and Psychotherapy, Faculty of Medicine and University Hospital Cologne, University of Cologne, Cologne, Germany

**Keywords:** Psychometrics, Clinical interviews, Semi-structured interviews, Symptom severity, Children and adolescents, Internalizing and externalizing symptoms

## Abstract

**Background:**

This study evaluates the psychometric properties of the newly developed semi-structured interview, Interview Version of the Symptoms and Functioning Severity Scale (SFSS-I), which is designed to provide a dimensional assessment of internalizing and externalizing symptoms.

**Methods:**

Multi-informant baseline data from the OPTIE study was used, involving 358 children and adolescents aged 6 to 17 years (*M* = 11.54, *SD* = 3.4, *n* = 140 [39.1%] were female). Participants were screened for internalizing and externalizing symptoms. For validity analyses, caregiver (Child Behavior Checklist), youth (Youth Self Report), and teacher ratings (Teacher Report Form) were used. We performed Receiver Operating Characteristic (ROC) analyses to evaluate the effectiveness of the SFSS-I subscales in distinguishing between children and adolescents diagnosed with internalizing and externalizing disorders, as determined by clinical judgement in routine care.

**Results:**

Confirmatory factor analyses supported a correlated two-factor model for internalizing and externalizing symptoms. Acceptable to good internal consistencies (α = 0.76 to 0.89; ω = 0.76 to 0.90) and excellent interrater reliability on the scale level (ICC ≥ 0.91) was found. The ROC analyses showed an acceptable accuracy in identifying internalizing diagnoses (AUC = 0.76) and excellent accuracy for externalizing diagnoses (AUC = 0.84).

**Conclusion:**

The SFSS-I demonstrates potential as a clinically-rated instrument for screening and routine outcome monitoring, offering utility in both clinical practice and research settings for the dimensional assessment of broad psychopathological dimensions.

**Trial registration:**

German Clinical Trials Register (DRKS) DRKS00016737 (https://www.drks.de/DRKS00016737). Registered 17 September, 2019.

**Supplementary Information:**

The online version contains supplementary material available at 10.1186/s13034-024-00788-y.

## Introduction

For the assessment of mental disorders in children and adolescents, clinical interviews are available in unstructured, semi-structured, and fully structured form [1–5]. These interviews were developed to enhance the reliability and validity of psychological assessments and to reduce diagnostic discrepancies caused by factors, such as information- and interpretation variance, criterion variance, and other heuristics [4–7]. Primarily, clinical interviews are used in research, but have also become more common in clinical settings [2, 7].

Structured clinical interviews often follow a categorical approach, mostly based on criteria of the Diagnostic and Statistical Manual of Mental Disorders (DSM) [8] or the International Statistical Classification of Diseases and Related Health Problems (ICD) [9], where a clear line is drawn between normal and abnormal behavior [2, 3, 10]. However, this approach may not fully capture the complexity of mental disorder, as symptom severity can vary among patients, and those with subclinical symptoms may still experience significant impairment, even if they do not meet the predefined diagnostic criteria [10, 11]. Examples of categorical clinical interviews include the Child and Adolescent Psychiatric Assessment (CAPA) [12], the Children’s Interview for Psychiatric Syndromes (ChIPS) [13], and the Diagnostic Interview for Children and Adolescents (DICA) [14].

In contrast, a dimensional diagnostic approach aims to capture a more nuanced picture of symptom severity by considering the different levels of symptoms across individuals. Although the dimensional assessment of mental health has gained increasing recognition in recent years, there is an ongoing debate about whether mental disorders should be classified categorically, dimensionally (symptoms are rated along a continuum), or through a combination of both [15–19]. The availability of clinical interviews for children and adolescents that include scales that allow for symptom severity to be measured along a continuum remains limited, and includes the German DISYPS-ILF [20–22], the Semistructured Clinical Interview for Children and Adolescents (SCICA) [23], and/or the Anxiety and Related Disorders Interview Schedule for DSM-5, Child and Parent Version (ADIS-5) [24, 25].

Furthermore, in recent years, research has increasingly suggested that a limited number of core factors may under lie the diverse range of mental disorders observed, indicating that many different disorders may share common underlying mechanisms [10, 18, 26, 27]. Although the transdiagnostic perspective was not developed at that time, Achenbach [28] proposed an internalizing and externalizing dimension, recognizing early on that different symptoms could be attributed to only a few general factors. The internalizing factor may include symptoms associated with depression, anxiety, or post-traumatic stress disorder, while the externalizing factor may include outwardly displayed symptoms related to substance abuse-, hyperactive-, disruptive-, impulsive-, and antisocial-related disorders [10, 28, 29]. The internalizing-externalizing model has been replicated in different studies on transdiagnostic comorbidity research with diverse populations [26, 28, 30–32]. However, to the best of our knowledge, the SCICA is the only clinical interview available with psychometrically evaluated scale scores for both internalizing and externalizing behaviours.

Most standardized clinical interviews currently available for assessing mental health problems in children and adolescents are typically categorical and disorder-specific. In contrast, our study aimed to develop a semi-structured interview called SFSS-I, which, to our knowledge, combines features that none of the existing instruments do: (i) Consideration of two reliably identified transdiagnostic dimensions, namely, internalizing and externalizing symptoms, which are commonly represented in children and adolescents. (ii) A dimensional assessment approach to measure varying degrees of psychopathology. (iii) A tool suitable for research or practical settings, particularly useful when obtaining reliable data from very young children through self-rating scales is challenging, or in more complex cases. The ADIS-5 and the DISYPS-ILF are both disorder-specific interviews based on the DSM-5 [8] and/or ICD-10 [33]. The DISYPS-ILF relates to the internalizing-externalizing model concept; however, currently, it does not offer psychometrically validated scale scores for these categories. Furthermore, in terms of the number of items, neither the SCICA (≥ 113 items) nor the DISYPS-ILF (74 items for externalizing disorders, 95 items for internalizing disorders) meets the criterion for a practical tool.

The SFSS-I’s development was based on two established instruments for assessing mental health problems in children and adolescents. The items were taken from the Symptoms and Functioning Severity Scale (SFSS) [34, 35] and the interview format was adapted from the German DISYPS-ILF [20]. The SFSS included in the Peabody Treatment Progress Battery (PTPB) [35] is a comprehensive system designed for feedback-informed treatment. The SFSS is similar to the Child Behavior Checklist (CBCL) [36], but contains only up to 27 items. Even though the model fit for a two-factor solution was not optimal (clinician form: comparative fit index (CFI) = 0.82, Jörekskog’s goodness of fit index (GFI) = 0.79, standardized root mean square residual (SRMR) = 0.007) [35], several studies have supported the reliability and validity of the internalizing and externalizing domains [34, 35, 37–39]. We chose the SFSS due to its brevity and its ability to provide a dimensional assessment of two common transdiagnostic subscales. We adapted the rating format of the German DISYPS-ILF because its items are similar to the SFSS and its manual provides detailed information for the exploration and scoring of each item, which is crucial for rating reliability and validity. In preliminary psychometric analyses of the German DISYPS-ILF regarding externalizing disorders (e.g., attention-deficit/hyperactivity disorder, oppositional defiant disorder), we found good psychometric properties [21].

The interview version of the SFSS (SFSS-I) was developed as part of the German OPTIE study (Optimizing treatment outcomes through progress feedback in cognitive behavioral therapy for children with internalizing and externalizing disorders) [40] and serves as the primary outcome parameter in the trial. The SFSS-I primarily focuses on symptom severity, which is assumed to be associated with other important clinical outcomes. For example, a strong and positive relationship is assumed between symptom severity and functional impairment [41]. However, a systematic review demonstrated that the average of the 497 correlations across symptomatology of different anxiety disorders and diverse functional impairment domains (e.g., physical, social) was only moderate (*r* = 0.34). Additionally, a comparable review on depression also revealed a moderate correlation between symptom severity and functional impairment [42]. Together, these reviews challenge the assumption that symptom severity is strongly and positively correlated with impaired functioning and indicate that the relationship is more complex.

We expanded the original brief questionnaire, SFSS, into a more comprehensive interview for two primary reasons. First, the interview served as an added measure to enhance reliability. Secondly, because the research staff had only limited contact with the family due to the nature of the study, this extended format ensured we gathered all essential information for accurate rating. The main aim of the current study was to report the development of the instrument and to evaluate the psychometric properties of the SFSS-I using baseline data from the OPTIE study sample. The psychometric evaluation included (1) descriptive statistics, (2) factor structure, (3) reliability (internal consistency and interrater reliability), (4) total-item correlations, (5) convergent and discriminant validity, and (6) receiver operating characteristic (ROC) analysis.

## Methods

### Participants and procedure

Participants were drawn from the OPTIE study that investigated the efficacy of feedback-informed treatment in behavioral therapy for children and adolescents [40]. The evaluation of the SFSS-I was based on data from 358 families at baseline who fulfilled the following criteria: (i) were eligible for the OPTIE study, (ii) provided informed consent for the participation in the study, (iii) the interview was conducted at baseline with the family, and (iv) were diagnosed with at least one psychological disorder [33].

The recruitment process for the OPTIE study took place from September 2019 to November 2022 at the outpatient unit of the School for Child and Adolescent Psychotherapy (AKiP) in Germany. The outpatient unit provides behavioral therapy for children and adolescents and is part of the general health care provided in Germany. As part of the routine screening procedure for families who applied for behavioral psychotherapy, eligibility for the OPTIE study was determined based on the following criteria: (i) patients 6 to 17 years old, (ii) presence of internalizing and/or externalizing symptoms based on a clinical rating of a senior psychotherapist who screened the patient, (iii) indication for outpatient behavior psychotherapy based on a clinical judgment by the screener, and (iv) at least one caregiver who speaks and understands German to a sufficient degree. After determining family’s eligibility, the SFSS-I was conducted with the primary caregiver, which was audio recorded with their consent.

The OPTIE study adhered to the ethical standards outlined in the Declaration of Helsinki [43] and the code of conduct of the Federal Chamber of Psychotherapists in Germany [44]. The study was approved by the Ethic Commission of the Medical Faculty of the University of Cologne (ID 18-435). For the participation in the study, written informed consent was obtained from both caregivers (if both had custody of the child), and the children and adolescents signed a declaration of intent.

### Measures

In this analysis, all measures were assessed at the baseline of the OPTIE study, which included two types of diagnostic measure: interview data and questionnaire data.

#### Interview Version of the Symptoms and Functioning Severity Scale (SFSS-I)

##### Items

The items for the interview were taken from the SFSS which is part of the measurement feedback system PTPB [35]. The SFSS has forms available for youths, caregivers, and clinicians, and can be used regularly with short time-intervals to provide information about the general symptom severity. In its original form, the SFSS has up to 27 items. We considered only those 24 items that are used for total scale computation and excluded the remaining three items that are included in the instrument for clinical purpose only (e.g., use drugs non-medical). Three scale scores can be computed: a Total score (24 items) and the two subscale scores Internalizing (10 items) and Externalizing (14 items). For the study, the items of the SFSS-I were reordered and therefore differ from the item order of the original SFSS. Items 1 to 14 reflected all externalizing items and Items 15 to 24 reflected all internalizing items.

##### Interview and rating format

The interview and rating format for the SFSS-I was adapted from the German DISYPS-ILF [20], which is a semi-structured interview that allows both categorical diagnoses according to DSM-5 and dimensional assessment [e.g., 21, 22] provides the following information for each item: (i) a description of behaviors and feelings meant to be assessed by each item, (ii) possible questions for exploration, and (iii) for each rating score a short description of the intended symptom severity. During the development process of the SFSS-I, corresponding information was provided for each item (see Supplemental Table S1). Clinicians rated symptoms that had been observed during the last six weeks. For the sake of consistency, we adopted the 4-point Likert scale from the DISYPS-ILF ranging from 0 (*not at all/age-typical*) to 3 (*very much*) instead of the 5-point Likert scale from the SFSS. A higher SFSS-I score indicates higher symptom severity. According to the DISYPS-ILF manual, item scores of two and higher are considered as clinically relevant. To calculate the scale scores, the corresponding items scores of each scale were summed.

##### Item translation procedure

For the OPTIE study, the items of the SFSS were translated into German and adapted for this context (see Supplemental Table S2). The procedure was based on translation and adaptation guidelines from the International Test Commission [45] and the Programme for International Student Assessment [46]. The aim was to adapt the item translation to the cultural context of the target language and to retain the grammatical structure as far as possible. After the initial translation of the original 24 SFSS items into German by a bilingual research assistant, the translation was discussed and compared with the original version on the item level by an expert panel consisting of four researchers with a clinical background and proficient understanding of the German and English languages. For certain items, a conscious decision was made to deviate slightly from the original translation to improve the comprehensibility and the differentiation from other items. For example, for Item 18 the word “physical” was added and the text was changed to “Gets into physical fights with family/friends” to clarify the exact meaning and to distinguish the content from Item 8 “Argues with adults”. Subsequently, two research assistants independently translated the modified German version back into English to check for the congruency of the content with the original SFSS items. The two translations were discussed again in the expert panel to identify further possible discrepancies. A final translation of the SFSS-I [47] was chosen that was congruent to the German modified vision and mostly congruent with the originally SFSS items (see Supplemental Table S2). Afterwards, a manual was written in German that included general instructions on how to conduct the interview and additional information for exploring the items. In a final step, a bilingual research assistant also translated the German interview manual into English.

##### Interview training

All interviewers were staff members of the OPTIE study. Before the SFSS-I was used, all interviewers took part in interview training to learn how to apply the interview and score the items. The training included an introduction to the conceptual basis of the interview, the presentation of pre-recorded audio files, test ratings, and feedback on the ratings. Additionally, all interviewers observed an interview trainer conducting the interview and subsequently rated the items on their own. Furthermore, regular calibration meetings were held to ensure the adequate implementation of the interview.

#### Achenbach System of Empirically Based Assessment (ASEBA) school-age forms & profiles (CBCL/6–18R, YSR/11–18R, TRF/6–18R)

To investigate the convergent and discriminant validity of the interview, we used the Internalizing and Externalizing Problems scales of the Child Behavior Checklist for Children Ages 6–18 (CBCL/6–18R) rated by caregivers, the Youth Self-Report (YSR/11–18R) rated by children of 11 years and older, and the Teacher’s Report Form (TRF/6–18R) rated by teachers [36, 48]. The scales were chosen because they (i) consider comparable constructs and time intervals (the last six months), (ii) are widely used, and (iii) have good psychometric properties. The internal consistency for both broad spectrum scales in all three forms showed good to excellent reliability in a German clinical and community sample [α > 0.80; 48] as well as in our sample (α ≥ 0.87).

### Statistical analysis

All statistical analyses were performed using SPSS and R. Missing data from questionnaires were imputed at the scale level, while missing data from interviews and demographics were imputed at the item level by the R package missForest [49]; for detailed information see result section. Imputed questionnaire values with decimals resulting from the imputation process were not further adjusted by rounding or truncation, as the original questionnaire data only allows integers, to prevent potential bias [50]. All SFSS-I items were checked for normal distribution as well as for floor effects, and ceiling effects based on skewness and kurtosis [51, 52].

At the beginning of the study, we also considered conducting an exploratory factor analysis (EFA) to investigate the dimensional structure of the data. However, we ultimately decided against this due to the clear hypotheses that could be formulated based on the conception and confirmatory factor analysis (CFA) results of the original instrument, as well as the risk of small sample size if the total sample (*N* = 358) was split.

#### CFAs

In the present study, whether a correlated two-factor model with an internalizing and an externalizing domain could be confirmed by CFA was systematically evaluated. For parameter estimation, the weighted least squares means and variance adjusted estimator (WLSMV) was chosen as the items were ordered categorically and because the estimator does not make distributional assumptions regarding the observed items [53]. To evaluate the model fit, the following indicators were assessed: the chi-square test (χ²), CFI, the Tucker–Lewis index (TLI), the root mean square error of approximation (RMSEA), and SRMR. Model fit was interpreted as acceptable when RMSEA or SRMR were ≤ 0.08 and as good when they were ≤ 0.05. CFI and TLI were considered as acceptable when they were ≥ 0.90 and as good when they were ≥ 0.95 [54, 55]. Because the chi-square test is sensitive to sample size, more emphasis was put on the other indicators. Furthermore, we calculated the Akaike information criterion (AIC) and the Bayesian information criterion (BIC) by using the maximum likelihood estimation with robust standard errors (MLR) to compare the different factor models (smaller values are preferred).

#### Interrater reliability

##### Subsample for the analysis

To assess the interrater reliability of the SFSS-I, we randomly selected a subsample of *n* = 61 audiotaped interviews that were originally conducted by three different trained interviewers. The sample size was calculated based on guidelines for interrater reliability studies, which recommend using the intraclass correlation coefficient (ICC) [56]. We took several parameters into account: a required minimum value ρ_0_ for the ICC value that is pre-specified to be acceptable; an expected value ρ for the ICC that is not less than ρ_0;_ the number of ratings for each patient (*k*); the desired power; and alpha [57, 58]. Under the assumption of ρ = 0.75, ρ_0_ = 0.60, *k* = 3, tails = 1, power = 0.80, and alpha = 0.05, a sample size of a minimum of 53 interviews was needed for a one-sided test [57, 58]. We decided to select eight additional audio recordings of interviews (around 15%) in case of poor sound quality or technical issues.

##### Rating procedure

The selected audiotaped interviews were rated by two additional raters who had prior experience of conducting interviews and were blind to the original rating scores and the treatment group of the OPTIE study [40]. Before starting the rating procedure, both raters participated in a short workshop to refresh their knowledge and rated four randomly selected practice audio recordings to ensure consistent understanding of the items.

##### Score computation

For the interrater reliability, as the symptoms of each selected patient (*n* = 61) were rated a total of three times, by the original interviewer and two additional raters (*k* ≥ 2), giving the continuous data type on a scale level, the interrater reliability was computed by the ICC [57, 59, 60]. Additionally, despite that fact that on the item level the rating scale was ordered categorically, the ICC was also computed for this data type to facilitate comparisons. For the ICC calculation there are several options regarding the model (one-way random-effects, two-way random-effects, or two-way fixed-effects), the type (single rater/measurement or mean of *k* raters/measurement), and the definition (absolute agreement or consistency). For the analysis, the one-way random-effects, absolute agreement model for single rater ICC(1,1) was chosen, for the following reasons. First, the one-way random-effects model was chosen because it was not possible for all baseline/original interviews to be conducted and rated by the same interviewer. Second, the single rater type was selected because the rating should be based on the judgment of one interviewer and not based on all interviewers; however, for comparison with other studies, we also calculated the ICC(1,3) for the mean value of three raters. Third, the absolute agreement model was decided on to check if multiple interviewers would rate the same information in exactly the same way on the scale level and on the item level [61]. Additionally, the ICC was calculated only among the two additional raters (*k* = 2) who both rated all of the 64 selected audio recordings, and for this purpose the two-way random-effects model, absolute agreement for single rater ICC(2,1) and for the mean value of 2 raters ICC(2,2) was chosen. There are no standard values for the interpretation of the ICC; however, researchers often indicate ICC values ≤ 0.50 as poor, values between 0.50 and 0.75 as moderate, values between 0.75 and 0.90 as good, and values ≥ 0.90 as excellent [61].

#### ROC analysis

An ROC analysis was conducted to determine the ability of the SFSS-I Internalizing and Externalizing subscales (test variables) to differentiate between children and adolescents with and without an internalizing or externalizing diagnosis (state variables) and to determine an optimal cut-off score for diagnosis per subscale. As part of the routine care process, the primary diagnosis was assigned by the child’s psychotherapist. All diagnoses were based on the clinical judgement of the psychotherapist according to the ICD-10 criteria [33] and in most cases, the assigned diagnosis was validated with a German clinical, disorder-specific diagnostic checklist [62]. For the purpose of the ROC analyses, the primary diagnosis was classified as an internalizing, externalizing, or other diagnosis (see Supplemental Table S3). The ROC curve is a graphical representation of the relation between the true positive rate (sensitivity, y-axis) and the false positive rate (1 − specificity, x-axis) for different classification thresholds of the SFSS-I subscales. The area under the curve (AUC) is the area under the ROC curve and is a statistical indicator of the performance of a binary classification model. The larger the AUC, the better the discrimination [63]. AUC scores can range from 0.50 (at random) to 1 (perfect), and the following interpretation was used: 0.50 ≤ AUC < 0.70 poor, 0.70 ≤ AUC < 0.80 acceptable, 0.80 ≤ AUC < 0.90 excellent, 0.90 ≤ AUC outstanding [64]. Additionally, the Youden Index was employed to determine the optimal cut-off scores for the Internalizing and Externalizing subscales. The index is defined as sensitivity + specificity– 1 and is calculated by finding the point on the ROC curve that maximises the distance between the line of equality (AUC = 0.50), where sensitivity equals specificity. The highest Youden value identifies the best cut-off. The Youden Index ranges from 0, indicating a test that performs not better than random chance (50% sensitivity and 50% specificity), to 1, indicating a test with perfect performance (100% sensitivity and 100% specificity) [65].

To provide information about the clinical utility of the proposed cut-off scores of the SFSS-I, measures, such as positive/negative likelihood ratios, positive/negative predictive values, and diagnostic accuracy were calculated. We also calculated the changes in pre- to post-test probabilities for the presence and absence of internalizing and externalizing disorders using the proposed cut-off scores. The positive likelihood ratio is the ratio of the probability of obtaining a positive test result (here: subscale score equal to or higher than identified cut-off score) if diagnosed with a disorder to the probability of obtaining a positive test result if not diagnosed. The negative likelihood ratio is the ratio of the probability of obtaining a negative test result (here: subscale score below the identified cut-off score) if diagnosed to the probability of obtaining a negative test result if not diagnosed. Additionally, the positive predictive value refers to the probability that a disorder is present when the subscale score is equal to or higher than the identified cut-off score of the instrument. Conversely, the negative predictive value refers to the probability that a disorder is not present when the scale score is below the identified cut-off score. Furthermore, diagnostic accuracy refers to the ability of the diagnostic instrument to correctly classify children and adolescents into subgroups based on whether they meet the criteria for a diagnosis [66].

## Results

Overall, the interview data were very complete and only in Items 1, 15, and 19 did one value have to be replaced. For caregivers, adolescents, and teachers there were 33 (9.22%), 22 (12.43%), and 130 (36.31%) questionnaires missing, respectively, and values were imputed on the scale level. The demographic data showed missing values for the following variables (number of missing cases and percentages in parentheses): highest guardian’s education (91; 25.42%), relationship status parents (11; 3.10%), psychological disorder(s) in family (85; 23.74%), ICD category (6; 1.68%), and pre-treatment(s) (21; 5.87%).

Demographic and diagnostic information for the total sample and for the interrater sub-sample are provided in Table 1 (see also Supplemental Table S4). The time it took to conduct the SFSS-I at baseline varied depending on the number and severity of the symptoms of the child/adolescent but, on average, it took about 50 min to complete. The interview was conducted with the biological mother in 304 cases (85%), with the biological father in 40 cases (11%), and in 14 cases (4%) with another caregiver (e.g., grandparents, adoptive parents, foster parents).

**Table 1:** Baseline characteristics

Baseline characteristics	Total sample *N* = 358	Subsample IRR *n* = 61
Child demographics		
Sex, female, *n* (%)	140 (39.1)	23 (37.7)
Age, *M* (*SD*)	11.54 (3.4)	11 (3.0)
Youth, age ≥ 11 years, *n* (%)	177 (49.4)	25 (41.0)
Screening results, *n* (%)^a^		
Internalizing symptoms	238 (66.5)	32 (52.5)
Externalizing symptoms	194 (54.2)	43 (70.5)
ICD no. of diagnoses, *n* (%)		
No	6 (1.7)	0 (0)
1	188 (52.5)	37 (60.7)
2	109 (30.4)	13 (21.3)
3	55 (15.4)	11 (18.0)
ICD main category, *n* (%)		
Internalizing	168 (46.9)	20 (32.8)
Externalizing	143 (39.9)	33 (54.1)
Others	47 (13.1)	8 (13.1)
Pre-treatment(s), *n* (%)	254 (70.9)	45 (73.8)
Guardian/family variables		
Highest guardians’ education, *n* (%)		
No/primary education	1 (0.3)	0 (0)
Lower secondary education	126 (35.2)	27 (44.3)
Post-secondary non-tertiary education	117 (32.7)	18 (29.5)
Tertiary education	114 (31.8)	16 (26.2)
Relationship status parents, *n* (%)		
Live together	236 (65.9)	41 (67.2)
Separated (others)	122 (34.1)	20 (32.8)
Psychological disorder(s) known within the family, *n* (%)	202 (56.4)	38 (62.3)

The classification of the highest education is based on the International Standard Classification of Education [67]

^a^True rates (%) of internalizing and externalizing symptoms. The total percentage exceeds 100% due to 74 (total sample)/14 (subsample IRR) participants having both kind of symptoms (internalizing and externalizing)

### CFA

To evaluate the factor structure of the SFSS-I, we tested and compared four CFA models: (1) a one-factor model, (2) an uncorrelated as well as (3) a correlated two-factor model representing an externalizing and an internalizing dimension, and (4) a correlated four-factor model representing four symptom domains (hyperactivity/impulsivity, aggressive-dissocial behavior, depression, and anxiety).

**Fig. 1:**
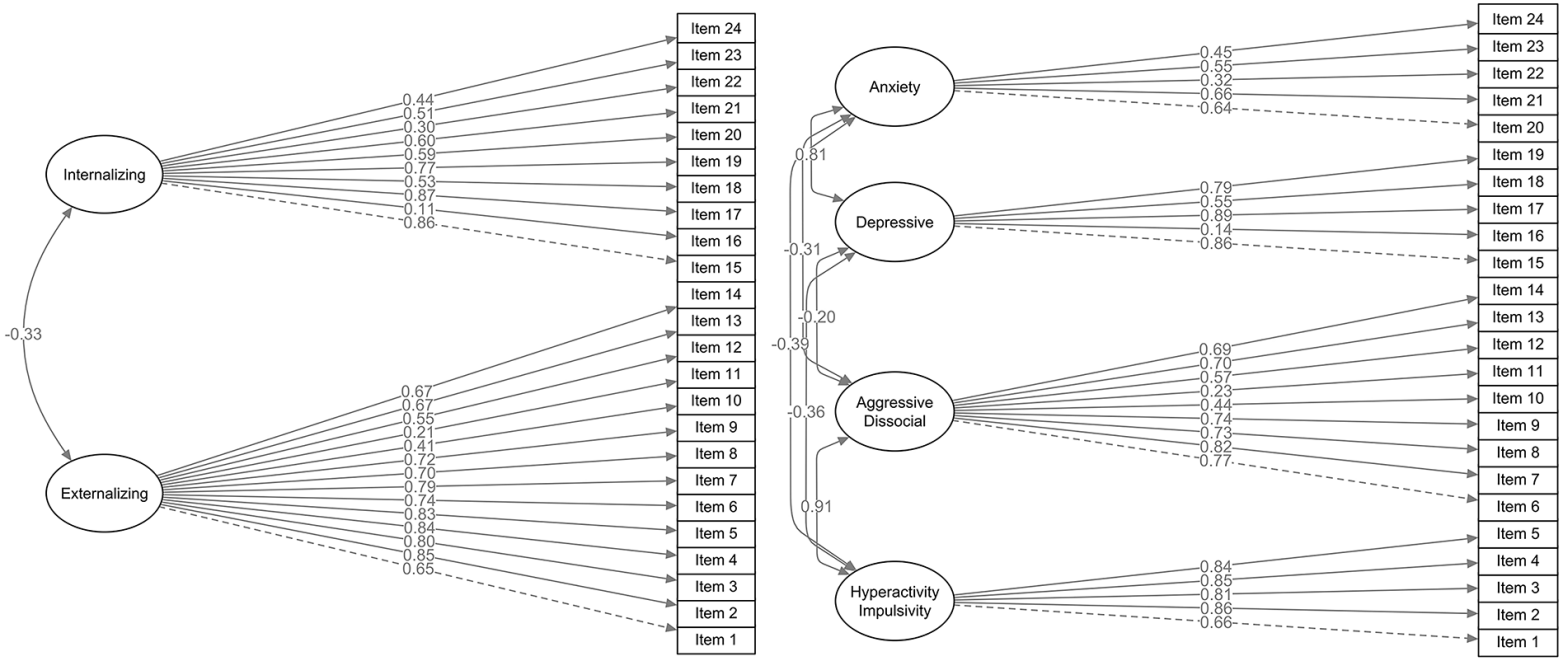
Correlated two-factor model and correlated four-factor model in the total sample (*N* = 358). *Note* Results are based on the weighted least squares means and variance adjusted estimator (WLSMV). The item order of the SFSS-I differs from that of the original SFSS instrument and items have been rearranged. All correlations and factor loadings were significant (*p*  < 0.05), except for the factor loading of Item 16 (*p*  = 0.07) of the correlated-two factor model

The one-factor and the uncorrelated two-factor models both showed a poor model fit (CFI, TLI ≤ 0.88; RMSEA, SRMR ≥ 0.09). Although the correlated four-factor solution showed slightly better model fit indices (CFI = 0.922, TLI = 0.912, RMSEA = 0.071, SRMR = 0.098, AIC = 20544.09, BIC = 20753.64; Table 2), the a priori proposed correlated two-factor model was preferred due to acceptable to good model fit indices, except for the SRMR (CFI = 0.917, TLI = 0.909, RMSEA = 0.072, SRMR = 0.103, AIC = 20626.70, BIC = 20816.84). The four-factor model had poor internal consistency for the anxiety factor (Items 20 to 24; α = 0.58; Supplemental Table S5). Additionally, the latent factors hyperactivity/impulsivity and aggressive-dissocial (*r* = 0.91), as well as depression and anxiety, were highly positively correlated (*r* = 0.81; see Fig. 1). Based on these results and the theoretical background, we concluded that the correlated two-factor model best captured our data. For the preferred correlated two-factor model, all standardized factor loadings (λ) were positive and λ > 0.41, except for Item 11 (λ = 0.21), Item 16 (λ = 0.11), and Item 22 (λ = 0.30; see Fig. 1). A significant moderate negative correlation (*r* =–0.33) was found between the two latent factors internalizing and externalizing.

**Table 2:** Goodness-of-fit statistics of the CFAs in the total sample (*N* *= *358)

Model	χ^2^	*df*	CFI	TLI	RMSEA	90% CI	SRMR	AIC	BIC
	WLSMV							MLR	
One factor	1707.11*	252	0.74	0.71	0.13	[0.12, 0.13]	0.15	21158.40	21344.66
Two factor (uncorrelated)	920.33*	252	0.88	0.87	0.09	[0.08, 0.09]	0.13	20648.38	20834.64
Two factor (correlated)	714.55*	251	0.92	0.91	0.07	[0.07, 0.08]	0.10	20626.70	20816.84
Four factor (correlated)	683.07*	246	0.92	0.91	0.07	[0.06, 0.08]	0.10	20544.09	20753.64

CFI, comparative fit index; TLI, Tucker–Lewis index; RMSEA, root mean square error of approximation; WLSMV, weighted least squares means and variance adjusted estimator; CI, confidence interval; SRMR, standardized root mean square residual; AIC, Akaike information criterion; BIC, Bayesian information criterion; MLR, maximum likelihood estimation with robust standard

*p < 0.001

### Item and scale characteristics

Descriptive statistics did show that the SFSS-I items were normally distributed except for Items 10 (“Gets into trouble for his/her rule breaking or antisocial behavior”), 11 (“Spends time with other children/youth who do not follow rules or are antisocial”), and 14 (“Threatens or bullies others”), which were answered with 0 (*not at all/age-typical*) in ≥ 82% of the cases (see Supplemental Table S6). Item-total correlations of the internalizing and externalizing domains were acceptable (0.36 ≤ *r*_it_ ≤ 0.72) with exceptions for the Items 10, 11, 16, and 22 (*r*_it_ < 0.30; see Table 3). Item-total correlations of the Total scale were generally acceptable for the first 14 items (0.39 ≤ *r*_it_ ≤ 0.49); however, Items 10 and 11 (*r*_it_ ≤ 0.27; see Table 3), as well as Items 15 to 24, had lower correlations (*r*_it_ ≤ 0.32; see Table 3). Nevertheless, in our preliminary analysis, we chose to retain items with low item-total correlations because we believed they held clinical relevance, and we aimed to maintain comparability with the original SFSS. It is also possible that different results may be obtained with a different clinical sample.

**Table 3:** Item total correlations (*N* *= *358)

Item	Item-total		
	Internalizing	Externalizing	Total
1. Finds it hard to pay attention or concentrate		0.55	0.40
2. Has a hard time waiting his/her turn		0.71	0.47
3. Finds it hard to sit still		0.64	0.39
4. Interrupts others		0.72	0.48
5. Has a flaring temper and difficulties controlling himself/herself		0.71	0.53
6. Throws things, when he/she loses his/her temper		0.56	0.42
7. Does not follow rules of adults		0.71	0.49
8. Frequently argues with adults		0.61	0.49
9. Gets into physical fights with family/friends		0.59	0.46
10. Gets into trouble for his/her rule breaking or antisocial behavior		**0.27**	**0.27**
11. Spends time with other children/youth, who do not follow rules or are antisocial		**0.17**	**0.20**
12. Lies to get something or gain advantages		0.47	0.35
13. Annoys others on purpose		0.58	0.51
14. Threatens or bullies others		0.41	0.34
15. Finds it hard to experience joy or have fun	0.51		**0.11**
16. Quickly starts to cry	**0.19**		0.32
17. Appears unhappy or sad	0.65		**0.17**
18. Feels worthless and has little self-confidence	0.48		**0.22**
19. Has little or no energy	0.55		**0.16**
20. Worries about a lot of things	0.48		**0.07**
21. Is nervous/shy	0.36		**–0.06**
22. Feels tense	**0.28**		**0.17**
23. Fears being laughed at	0.36		**0.09**
24. Has difficulties falling asleep or sleeping through or has other sleeping problems	0.43		**0.27**

The item order of the SFSS-I differs from that of the original SFSS instrument and items have been rearranged. Item-total correlations (*r*_*it*_) with values of > 0.30 considered acceptable. Bold values indicate items below the threshold

Descriptive statistics on scale level did show that the SFSS-I data was normally distributed. The internal consistencies measured by Cronbach’s alpha (α) and McDonald’s omega (ω) for the Internalizing, Externalizing, and the Total scale were mostly acceptable to good (α = 0.76 to 0.89; ω = 0.69 to 0.90; see Table 4).

**Table 4:** Scale characteristics of the SFSS in the total sample (*N* = 358)

Scale	*k*	*M*	*SD*	Skewness	Kurtosis	Min.	Max.	α	ω
Internalizing	10	9.17	5.67	0.54	–0.32	0	27	0.76	0.76
Externalizing	14	10.42	8.13	0.76	–0.08	0	38	0.89	0.90
Total	24	19.59	8.98	0.65	0.09	0	47	0.76	0.69

*k* = number of items; α = Cronbachs Alpha; ω = McDonald’s Omega.

### Interrater reliability

The interrater reliability of the SFSS-I scales for three ratings (interviewer, two additional raters) per participant (*k* = 3), as indicated by the ICCs(1,1) and (1,3), were excellent with ICC scores of ≥ 0.91 (see Table 5). In addition, if the interrater reliability was computed only for the two additional raters (*k* = 2), as indicated by the ICCs(2,1) and (2,2), excellent ICC scores of ≥ 0.91 were also obtained (see Supplemental Table S7). The interrater reliability, assessed on the item level by three ratings per participant (*k* = 3), showed moderate to excellent agreement, with ICC coefficients ranging from 0.53 to 0.97 (see Supplemental Table S8).

**Table 5:** Interrater reliability of the SFSS-I scales in the Subsample (*n* = 61)

Scale	ICC(1,1)	95% CI	ICC(1,3)	95% CI
Internalizing	0.95	[0.93, 0.97]	0.98	[0.97, 0.99]
Externalizing	0.91	[0.86, 0.94]	0.97	[0.95, 0.98]
Total	0.91	[0.86, 0.94]	0.97	[0.95, 0.98]

CI, confidence interval; ICC, intraclass correlation coefficient; ICC(1,1), one-way random-effects, absolute agreement model for single rater/measurements; ICC(1,3), one-way random-effects, absolute agreement model based on a mean-rating of one interviewer and two additional raters (*k* = 3)

### Convergent and discriminant validity

The SFSS-I Externalizing and Internalizing scales were compared to the corresponding scales of the ASEBA forms (see Table 6). The scales showed acceptable to good convergent validity (same construct) with caregiver (CBCL/6–18R; *r* = 0.68 to 0.72, *p* < 0.001), youth (YSR/11–18R; *r* = 0.28 to 0.43, *p* < 0.001), and teacher ratings (TRF/6-18R; *r* = 0.35 to 0.57, *p* <.001). Low correlations between the two SFSS-I scales and questionnaire scales that measure different constructs in caregiver (CBCL/6–18R; *r* =–0.18 to–0.12, 0.001 ≤ *p* ≤ 0.02), youth (YSR/11–18R; *r* =–0.27 to 0.05, 0.001 ≤ *p* ≤ 0.49), and teacher ratings (TRF/6–18R; *r* =–0.27 to–0.02, 0.001 ≤ *p* ≤ 0.66) further indicated good discriminant validity.

**Table 6:** Convergent and discriminant validity of the SFSS-I

	SFSS-I (clinician)	
	INT	EXT
CBCL (parents)		
INT	0.68***	–0.18***
EXT	–0.12*	0.72***
YSR (youth)		
INT	0.43***	–0.27***
EXT	0.05	0.28***
TRF (teacher)		
INT	0.35***	–0.02
EXT	–0.27***	0.57***

The total sample size is *N* = 358, except for the youth rating (YSR/11–18R, *n* = 177). SFSS-I = Interview version of the symptoms and functioning severity scale; CBCL/6–18R = Child behavior checklist 6-18R; YSR/11–18R = Youth self-report 11–18R; TRF/6–18R = Teacher’s report form 6–18R.; INT = Internalizing scale; EXT = Externalizing scale

*p < 0.05. **p < 0.01. ***p < 0.001

### ROC analysis

**Fig. 2:**
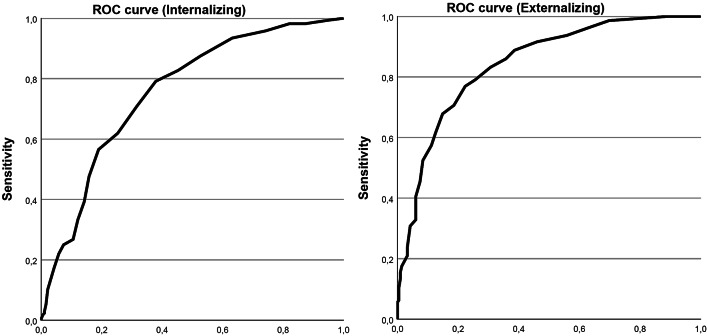
ROC curve of the Internalizing and Externalizing SFSS-I scale scores

In the ROC analysis, we investigated how well the two SFSS-I subscale scores could discriminate children with and without an internalizing and an externalizing diagnosis (see Fig. 2). Results demonstrated for the Internalizing scale an acceptable (AUC 0.76; 95% CI 0.71 to 0.81) differentiation accuracy and for the Externalizing scale an excellent (AUC 0.84; 95% CI 0.80 to 0.88) differentiation accuracy. Furthermore, the Youden Index indicated for the Internalizing scale an optimal cut-off point of 7.5 (sensitivity = 79.2%, specificity = 62.1%; see Supplemental Table S9) and for the Externalizing scale an optimal cut-off point of 10.5 (sensitivity = 76.9%, specificity = 77.7%; see Supplemental Table S9), meaning that these values would be best used as cut-off scores to indicate a potential diagnosis.

Additionally, the diagnostic utility of the SFSS-I was assessed for both the Internalizing and the Externalizing subscale. The identified SFSS-I cut-off scores correctly identified 79% (sensitivity) of the children with an internalizing diagnosis and 77% (sensitivity) with an externalizing diagnosis assigned by psychotherapists. Specificity was higher for externalizing disorders at 78% and for internalizing disorders at 62%. The positive likelihood ratio was calculated as 2.08 for internalizing disorders and 3.5 for externalizing disorders, suggesting that children with an internalizing diagnosis were approximately 2.08 times and with an externalizing diagnosis 3.5 times more likely to be classified positive on the SFSS-I than without a diagnosis. The negative likelihood ratio was 0.34 for internalizing disorders and 0.29 for externalizing disorders, indicating that participants without these diagnoses were less likely to be misclassified as having them by factors of 0.34 and 0.29, respectively. The positive predictive value was 65% for internalizing and 70% for externalizing, indicating the probability of a true diagnosis with scores at or above the cut-off score. The negative predictive value was 77% for internalizing and 85% for externalizing, indicating the probability of a true non-diagnosis with scores below the cut-off score. Additionally, the post-test probability of having an internalizing disorder increased by 17.9% points when using the proposed cut-off score of 7.5, and the probability of having an externalizing disorder increased by 29.6% points with a cut-off score of 10.5. Conversely, the post-test probability of not having an internalizing disorder decreased by 23.8% points, and the probability of not having an externalizing disorder decreased by 23.6% points when using the respective cut-off scores. Overall, diagnostic accuracy, which refers to the proportion of all cases that were correctly classified by the cut-off scores of the SFSS-I, was 70% for the Internalizing subscale and 77% for the Externalizing subscale (see Supplemental Tables S10–S14).

## Discussion

The aim of the study was to evaluate the psychometric properties of the German SFSS-I, a newly developed semi-structured clinical interview, intended for the dimensional assessment of symptom severity for both internalizing and externalizing symptoms in clinically-referred children and adolescents aged 6;0 to 17;11 years. In general, our results indicate that the SFSS-I is a reliable and valid measure.

We aimed to determine the optimal number of factors needed to describe the content of the items. In previous research, Bickman et al. [35] proposed a correlated two-factor solution that differentiated between internalizing and externalizing domains. Our analysis evaluated the fit of an unidimensional model, representing a broad general factor for psychopathology [68], an uncorrelated two-factor model, the preferred and validated correlated two-factor solution, and a newly tested correlated four-factor solution, based on four common psychological disorders in children and adolescents (attention-deficit/hyperactivity disorder, conduct/oppositional disorder, depression, and anxiety). We preferred the correlated two-factor model due to its overall better psychometric qualities across several measures (CFA, item-total correlation, reliability).

For this model, we found a significant negative small to moderate correlation between the internalizing and externalizing factor (*r* =–0.33). These results suggest that higher externalizing symptoms were more likely to co-occur with lower internalizing scores and vice versa. These findings were not in line with previous findings that showed a small to moderate positive correlation (*r* ≥ 0.19) between the two domains [35, 38, 48]. These discrepant findings may be due to differences in sample characteristics. For example, the children in our study may have fewer comorbidities (about 21% of the patients were screened with internalizing and externalizing symptoms), which could have influenced the observed relationships between the reported internalizing and externalizing symptoms.

For the two-dimensional externalizing-internalizing model, some items were only weakly related to their assumed dimension as indicated by low factor loadings and item-total correlation. This concerned the externalizing Items 10 (“Gets into trouble for his/her rule breaking or antisocial behavior”) and 11 (“Spends time with other children/youth who do not follow rules or are antisocial”) and the internalizing Items 16 (“Quickly starts to cry”) and 22 (“Feels tense”). Several factors may have contributed to these findings. For example, Bickman, Athay [35] found similar results for Item 11. With respect to Item 16, we believe that the instructions in the SFSS-I manual may have been misleading. Rather than focusing on only internalizing behaviors, interviewers were instructed to also explore whether children start to cry when a ban is imposed, which may reflect situations more relevant to externalizing behaviors. To better capture internalizing behaviors, we suggest a reformulation of the exploration questions for Item 16 in the interview guidelines.

We further assessed the degree of agreement on the SFSS-I among an interviewer and two independent raters. Most studies on (semi-)structured clinical interviews for children and adolescents do not conduct IRR-analyses based on a blind rating as was done in this study. Results showed excellent agreement on scale level (ICC ≥ 0.91) and moderate to excellent agreement on item level (ICC = 0.53–0.97), not only demonstrating that different raters were rating the same symptom severity of patients, but also showing that small discrepancies between the raters still can occur. The following reasons may be responsible for possible small discrepancies among raters in our study: first, although interview training was conducted beforehand and an interview guideline was used, it was evident that some raters were more thorough in exploring symptom frequency, severity, and contextual factors than others. In some cases, this may have resulted in those less thorough raters lacking important information necessary for accurate symptom assessment. Second, rater disagreement may also have occurred due to differences in interpretation of the statements of the caregivers (interpretation variance) [7].

Additionally, evidence was found for the convergent validity of the SFSS-I, indicating that the interview results was associated with other instruments intended to measure the same construct. The highest correlations were found between the corresponding SFSS-I scales and the parent rating (*r* = 0.68 to 0.72), with somewhat lower scores for the youth rating (*r* = 0.28 to 0.43) and the teacher rating (*r* = 0.35 to 0.57). Other studies also found low to moderate levels of agreement among different informant perspectives [e.g., caregivers, youth, teachers; 48, 69, 70], indicating that these values are normal and not specific to the SFSS-I. The strong association with the parent rating was in line with expectations, as parents were generally also the informant for the interview. In contrast, low correlations were found between the SFSS-I results and scales on questionnaires that were not intended to measure the same construct, demonstrating divergent validity. In general, our findings are consistent with other studies that have also examined the validity of interviews [1, 21].

Furthermore, our study found that the two subscales of the SFSS-I (Internalizing and Externalizing) effectively differentiated between children and adolescents diagnosed with internalizing or externalizing disorders and those without. In our study, the optimal scale cut-off score indicative of a disorder was (rounded) 8 for the Internalizing and 11 for the Externalizing scale. The SFSS-I, evaluated using the identified cut-off score, shows 62% specificity and 79% sensitivity for internalizing diagnoses, indicating that it is more accurate in detecting internalizing diagnoses than in ruling them out. For identifying or ruling out externalizing diagnoses, the identified cut-off score was about equally effective, with 78% specificity and 77% sensitivity. These results further indicate that the interview is about equally effective in detecting both internalizing and externalizing diagnoses, but more effective in ruling out externalizing disorders compared to internalizing disorders. This could be due to the differing ways children and adolescents express their symptoms. Caregivers may find it easier to rule out externalizing symptoms compared to internalizing symptoms during the interview as externalizing symptoms generally have a clearer appearance when present [71, 72]. The calculated changes in post-test probabilities, along with the likelihood ratios and predictive values, suggest the clinical relevance of the proposed cut-off scores. For both internalizing and externalizing disorders, the difference between base rates and post-test probabilities of the presence or absence of these disorders, while using the cut-off scores, exceeded 17% points. This can be considered a meaningful difference in clinical settings. Therefore, using the proposed cut-off scores can enhance diagnostic accuracy. However, further studies should also consider the use of multiple cut-off scores to indicate different levels of symptom severity (e.g., mild, moderate, severe).

One possible limitation of the study is that the interview was only conducted with the primary caregiver, whereas, due to cross-informant discrepancies, multiple perspectives should be considered [71, 73]; however, the interviewers did attempt to explore and assess the symptomatology across different contexts (e.g., at home, school). Results, such as a lower correlation between the SFSS-I and youth ratings (YSR/11–18R) or a rather small AUC value of the Internalizing scale, clearly indicate that it would be beneficial and important for further studies to develop parallel adolescent (≥ 11 years) and teacher versions [73]. This development could mitigate the potential loss of crucial information. Furthermore, another limitation is the lack of a uniform, standardized method for determining diagnosis, as the psychotherapists involved in this study relied on clinical judgment. This introduces a potential source of bias, as the subjective nature of clinical judgment can lead to variability in diagnosis, which can affect the reliability and replicability of the study’s findings. Therefore, future research should consider employing standardized diagnostic tools to enhance consistency and facilitate replication. Additionally, it was found that while the reliability scores for the Total scale were moderate to acceptable, multiple items (10, 11, and 14 to 24) had low item-total correlations (*r*_it_ ≤ 0.32), indicating weak relationships with the overall score. This was particularly evident for the internalizing items. We acknowledge that this could be a limitation when using the Total scale, and it may be partly explained by the unique characteristics of our sample, because in the correlated two-factor solution the externalizing and internalizing domain were negatively correlated, which is not consistent with previous studies [35, 38, 48]. Furthermore, data were analysed from a single sample of outpatient children and adolescents, and external validation is recommended. Future research should build on this study to investigate the psychometric properties of the SFSS-I in diverse samples (e.g., inpatient settings) to be able to generalize results. Additionally, future studies should examine the relationship between outcomes of the SFSS-I and SFSS. In comparison to the original-rated SFSS, the SFSS-I is more time-intensive. We see the potential application of the SFSS-I particularly in instances where a more thorough exploration is desirable and where the likely increase in reliability compensates for the extra time required.

## Conclusion

In conclusion, we obtained positive findings regarding the reliability and validity of the SFSS-I. We consider the SFSS-I as an accompaniment to existing interviews that have a focus on the extensive categorical assessment of specific mental disorders. In this semi-structured interview, with 24 items, that allows for a dimensional assessment while considering two reliably identified transdiagnostic factors (internalizing, externalizing) in clinically-referred children and adolescents to measure varying degrees of symptom severity, we see potential use of the measure in screening children and adolescents for a broad range of psychopathological symptoms in the field of routine care and research. The SFSS-I may prove particularly effective in both research and practical settings, especially when obtaining reliable data from very young children through self-reports is challenging, or in more complex cases.

### Supplementary Information

Supplementary Material 1.

## Data Availability

The raw data supporting the conclusions of this article will be made available by the authors upon reasonable request.

## Abbreviations

ADIS-5: Anxiety and Related Disorders Interview Schedule for DSM-5
AIC: Akaike information criterion
AKiP: School for Child and Adolescent Psychotherapy
ASEBA: Achenbach System of Empirically Based Assessment
AUC: Area under the curve
BIC: Bayesian information criterion
CAPA: Child and Adolescent Psychiatric Assessment
CBCL: Child Behavior Checklist
CFA: Confirmatory factor analysis
CFI: Comparative fit index
ChIPS: Children’s Interview for Psychiatric Syndromes
DICA: Diagnostic Interview for Children and Adolescents
DISYPS-ILF: Interview-Leitfäden zum Diagnostik-System für psychische Störungen nach DSM-5 für Kinder- und Jugendliche [Interview guidelines for the diagnostic system of mental disorders in children and adolescents based on DSM-5]
DRKS: Deutsches Register Klinischer Studien [German Clinical Trials Register]
DSM: Diagnostic and Statistical Manual of Mental Disorders
EFA: Exploratory factor analysis
ICC: Intraclass correlation coefficient
ICD: International Statistical Classification of Diseases and Related Health Problems
Jöreskog’s GFI: Jöreskog’s goodness of fit index
MLR: Maximum likelihood estimation with robust standard errors
OPTIE: Optimizing treatment outcomes through progress feedback in cognitive behavioral therapy for children with internalizing and externalizing disorders (study acronym)
PTPB: Peabody Treatment Progress Battery
RMSEA: Root mean square error of approximation
ROC: Receiver operating characteristic
SCICA: Semistructured Clinical Interview for Children and Adolescents
SFSS: Symptoms and Functioning Severity Scale
SFSS-I: Interview Version of the Symptoms and Functioning Severity Scale
SRMR: Standardized root mean square residual
TLI: Tucker–Lewis index
TRF: Teacher’s Report Form
WLSMV: Weighted least squares means and variance adjusted estimator
YSR: Youth Self-Report
