# Supplementary material for: A semi-structured interview for the dimensional assessment of internalizing and externalizing symptoms in children and adolescents: Interview Version of the Symptoms and Functioning Severity Scale (SFSS-I)
Source: Child Adolesc Psychiatry Ment Health. 2024 Aug 24;18:106. doi: 10.1186/s13034-024-00788-y (PMC11344912; doi:10.1186/s13034-024-00788-y)
Supplement: Supplementary file 1 — Supplementary Material 1. [file 13034_2024_788_MOESM1_ESM.docx]

**Supplementary Materials for:**

**A Semi-Structured Interview for the Dimensional Assessment of Internalizing and Externalizing Symptoms in Children and Adolescents: Interview Version of the Symptoms and Functioning Severity Scale (SFSS-I)**

*Child and Adolescent Psychiatry and Mental Health*

**Table S1**

*Example Items of the SFSS-I Guide*

| **Item** | **Scale** | **Exploration** | **Rating** |
| --- | --- | --- | --- |
| **Finds it hard to pay attention or concentrate** | Externalizing | **Interviewer:** Does __ find it hard to pay attention or concentrate?   - *Does he/she have a hard time staying attentive to one thing for a long time (e.g., homework, at school)? When he/she is playing or doing something specific, does he/she often stop or interrupt it because he/she gets distracted by all sorts of things (e.g. noise outside, other toys, own thoughts) or because he/she has thought of something else he/she would rather do? Does he/she make many careless mistakes, e.g., in homework? Does he/she seem not to listen when others talk to him/her?* - Areas of life  *In which areas of life/ situations does this (not) happen? (explore type and severity)* - in kindergarten? - at school? - at home? - on other occasions? - Frequency   In the past 6 weeks, how often did this happen in these situations? In more or less than half of the situations (*e.g., [almost] always – in most situations – in a few situations – rarely or never*)? How often do these problems not occur in such situations? | 0 not (worth mentioning) or age-typical  1 mild, e.g., in a few situations/on a few days per week  2 moderate, e.g., in several situations/on several days  3 very severe, e.g., almost always/every day  **Example of very severe symptomatology (3):**  Significantly reduced sustained attention (less than half of the duration that can be expected according to age) in many situations, e.g., in class, during conversations, while reading or playing, while watching television, or is distracted by even minor stimuli in almost all activities (e.g., by own thoughts about a later play date/appointment or when someone drives past the window, comes in the door, a bird chirps). Can only be controlled to a limited extent. |
| **Item** | **Scale** | **Exploration** | **Rating** |
| **Finds it hard to experience joy or have fun** *(Item 15)*  Focus is on **lack of positive affect**. Either because few positive activities exist or because there has been a loss of pleasure in otherwise positive activities.  **Differentiation**  **Item 16** *“Starts to cry quickly”*  Focus is on the **behaviour** of crying.  **Item 17** *“Seems unhappy or sad”*  Focus is on **negative affect** (e.g., feeling down, feeling empty).  **Item 19** *“Has little or no energy”*  Focus is on **drive** (e.g., being floppy, being exhausted). | Internalizing | **I**: Does ___ find it hard to experience joy or have fun?   - *What does this look like for him/her (e.g., most of the time he/she has no interest or enjoyment in almost everything he/she does, e.g., has no fun with peers, e.g., playing games, on trips? Also, no longer enjoys leisure activities that he/she once enjoyed, e.g., music, computers, television, sports?)* - Areas of life  *In which areas of life/situations does this (not) happen? (explore type and severity)* - in kindergarten (e.g., playing games)? - at school (e.g., playing at recess)? - at home (e.g., arranging to meet someone)? - on other occasions (e.g., going to the cinema, doing sports)? - Frequency   In the past 6 weeks, how often did this occur in these situations (*e.g., (almost) daily – 2 to 3 times per week – 1 time per week - rarely or never*)? How often do these problems not occur? | 0 not (worth mentioning) or age-typical  1 mild: e.g., in a few situations/approx. 1 time per week  2 moderate: e.g., in several situations/several times per week (approx. 2 to 3 times)  3 very severe: e.g., in many situations/(almost) daily  **Example of very severe symptomatology (3):**  Reacts to almost everything indifferently and listlessly. Does not enjoy activities and nice activities at home and elsewhere (e.g., does not enjoy hobbies, does not play much). |

*Note.* The item order of the SFSS-I differs from that of the original SFSS instrument and items have been rearranged.

**Table S2**

*Translation of SFSS-Items*

| Original Item | Literal translation to German | Modified German version | English translation of modified German version |
| --- | --- | --- | --- |
| Throw things when mad | Dinge werfen, wenn er/sie wütend ist | Wirft Gegenstände, wenn er/sie die Beherrschung verliert | Throws things, when he/she loses his/her temper |
| Feel unhappy or sad | Sich unglücklich oder traurig fühlen | Wirkt unglücklich oder traurig | Appears unhappy or sad |
| Get into trouble | In Schwierigkeiten geraten | Gerät durch sein/ihr regelbrechendes oder unsoziales Verhalten in Schwierigkeiten | Gets into trouble for his/her rule breaking or antisocial behavior |
| Have little or no energy | Wenige oder keine Energie haben | Hat wenig oder keine Energie | Has little or no energy |
| Disobey adults | Erwachsenen nicht gehorsam sein | Hält sich nicht an Regeln von Erwachsenen | Does not follow rules of adults |
| Interrupted others | Andere unterbrechen | Unterbricht andere | Interrupts others |
| Lie to get things | Lügen, um etwas zu bekommen | Lügt, um etwas zu bekommen oder um sich Vorteile zu verschaffen | Lies to get something or to gain advantages |
| Hard time controlling temper | Schwer das Temperament kontrollieren | Hat ein aufbrausendes Temperament und Schwierigkeiten, sich zu beherrschen | Has a flaring temper and difficulties controlling himself/herself |
| Worry about a lot of things | Sich über viele Dinge sorgen | Macht sich über viele Dinge Sorgen | Worries about a lot of things |
| Threaten or bully others | Andere bedrohen oder schikanieren | Bedroht oder schikaniert andere | Threatens or bullies others |
| Feel worthless | Sich wertlos fühlen | Fühlt sich wertlos und hat wenig Selbstvertrauen | Feels worthless and has little self-confidence |
| Have a hard time having fun | Schwierigkeiten damit Spaß zu haben | Fällt es schwer, Freude und Spaß zu haben | Finds it hard to experience joy or have fun |
| Feel afraid others laugh | Angst, dass andere (aus)lachen | Fürchtet sich, ausgelacht zu werden | Fears being laughed at/Is afraid of being laughed at |
| Hang out with kids in trouble | Zeit mit Kindern verbringen, die in Schwierigkeiten geraten | Verbringt Zeit mit anderen Kindern/Jugendlichen, die sich nicht an Regeln halten oder unsozial sind | Spends time with other children/youth, who do not follow rules or are antisocial |
| Feel nervous/shy | Sich nervös/schüchtern fühlen | Ist nervös/schüchtern | Is nervous/shy |
| Have a hard time paying attention | Schwierigkeiten damit aufmerksam zu sein | Fällte es schwer, aufzupassen und sich zu konzentrieren | Finds it hard to pay attention or concentrate |
| Get into fights with family/friends | In Streit mit Familien/Freunden geraten | Gerät mit der Familie/Freunden in körperliche Auseinandersetzungen | Gets into physical fights with family/friends |
| Have a hard time sitting still | Fällt es schwer, still zu sitzen | Fällt es schwer, ruhig sitzen zu bleiben | Finds it hard to sit still |
| Have a hard time sleeping | Schwierigkeiten beim Schlafen | Fällt es schwer ein- oder durchzuschlafen oder hat andere Schlafprobleme | Has difficulties falling asleep or sleeping through or has other sleeping problems |
| Feel tense | Sich angespannt fühlen | Fühlt sich angespannt | Feels tense |
| Cry easily | Schnell weinen | Fängt schnell an zu weinen | Quickly starts to cry |
| Annoy others on purpose | Andere absichtlich ärgern | Ärgert andere absichtlich | Annoys others on purpose |
| Argue with adults | Mit Erwachsenen streiten | Streitet häufig mit Erwachsenen | Frequently argues with adults |
| Hard time waiting turn | Schwierigkeiten damit abzuwarten bis er/sie dran ist | Fällt es schwer abzuwarten, bis er/sie an der Reihe ist | Has a hard time waiting his/her turn |

*Note.* The item order of the SFSS-I differs from that of the original SFSS instrument and items have been rearranged.

**Table S3**

*ICD-10 Diagnoses Classified according to the Categories (i) Internalizing, (ii) Externalizing, and (iii) Others (*N *= 358)*

| ICD-10 code | ICD-10 diagnosis | Category |
| --- | --- | --- |
| F32.0 | Mild depressive episode | internalizing |
| F32.2 | Severe depressive episode without psychotic symptoms | internalizing |
| F32.8 | Other depressive episodes | internalizing |
| F33.1 | Recurrent depressive disorder, current episode moderate | internalizing |
| F34.1 | Dysthymia | internalizing |
| F40.1 | Social phobias | internalizing |
| F40.2 | Specific (isolated) phobias | internalizing |
| F41.0 | Panic disorder [episodic paroxysmal anxiety] | internalizing |
| F41.1 | Generalized anxiety disorder | internalizing |
| F41.2 | Mixed anxiety and depressive disorder | internalizing |
| F41.3 | Other mixed anxiety disorders | internalizing |
| F41.9 | Anxiety disorder, unspecified | internalizing |
| F42.1 | Predominantly compulsive acts [obsessional rituals] | internalizing |
| F42.2 | Mixed obsessional thoughts and acts | internalizing |
| F43.1 | Post-traumatic stress disorder | internalizing |
| F43.2 | Adjustment disorders | internalizing |
| F43.25 | Adjustment disorders with mixed disturbance of emotions and conduct | others |
| F45.2 | Hypochondriacal disorder | internalizing |
| F45.3 | Somatoform autonomic dysfunction | internalizing |
| F45.4 | Persistent somatoform pain disorder | internalizing |
| F50.1 | Atypical anorexia nervosa | others |
| F50.2 | Bulimia nervosa | others |
| F60.31 | Emotionally unstable personality disorder: Borderline | internalizing |
| F63.3 | Trichotillomania | others |
| F63.8 | Other habit and impulse disorders | others |
| F63.9 | Habit and impulse disorder, unspecified | others |
| F64.0 | Transsexualism | others |
| F84.1 | Atypical autism | others |
| F84.5 | Asperger syndrome | others |
| F90.0 | Disturbance of activity and attention | externalizing |
| F90.1 | Hyperkinetic conduct disorder | externalizing |
| F90.8 | Other hyperkinetic disorders | externalizing |
| F91.0 | Conduct disorder confined to the family context | externalizing |
| F91.3 | Oppositional defiant disorder | externalizing |
| F91.8 | Other conduct disorders | externalizing |
| F91.9 | Conduct disorder, unspecified | externalizing |
| F92.0 | Depressive conduct disorder | others |
| F92.8 | Other mixed disorders of conduct and emotions | others |
| F93.0 | Separation anxiety disorder of childhood | internalizing |
| F93.1 | Phobic anxiety disorder of childhood | internalizing |
| F93.2 | Social anxiety disorder of childhood | internalizing |
| F93.3 | Sibling rivalry disorder | internalizing |
| F93.8 | Other childhood emotional disorders | internalizing |
| F95.0 | Transient tic disorder | others |
| F95.1 | Chronic motor or vocal tic disorder | others |
| F95.2 | Combined vocal and multiple motor tic disorder [de la Tourette] | others |
| F98.02 | Nonorganic enuresis nocturna et diurna | others |
| F98.1 | Nonorganic encopresis | others |
| F98.8 | Other specified behavioral and emotional disorders with onset usually occurring in childhood and adolescence | externalizing |

**Table S4**

*Rates of Categories (Internalizing/Externalizing/Others) for all ICD-10 Diagnoses (*N *= 358)*

| Category | *n* (%) |
| --- | --- |
| Only internalizing | 92 (25.7) |
| Only externalizing | 127 (35.5) |
| Only others | 18 (5.0) |
| Internalizing and externalizing | 47 (13.1) |
| Internalizing and others | 37 (10.3) |
| Externalizing and others | 27 (7.5) |
| Internalizing and externalizing and others | 10 (2.8) |

**Table S5**

*Internal Consistencies Four-Factor Model (*N *= 358)*

| Scale | Items | α |
| --- | --- | --- |
| Hyperactivity/Impulsivity | 1–5 | .86 |
| Aggressive - Dissocial | 6–14 | .79 |
| Depressive | 15–19 | .72 |
| Anxiety | 20–24 | **.58** |
| *Note.* α = Cronbach’s alpha. Bold values indicate items below the threshold of an acceptable internal consistency. | | |

**Table S6**

*Item Characteristics (*N *= 358)*

| Item | *Mean* | *SD* | Skewness | Kurtosis |
| --- | --- | --- | --- | --- |
| Externalizing |  |  |  |  |
| 1. Finds it hard to pay attention or concentrate | 1.61 | 1.11 | –0.18 | –1.32 |
| 2. Has a hard time waiting his/her turn | 0.82 | 1.05 | 0.86 | –0.70 |
| 3. Finds it hard to sit still | 1.16 | 1.21 | 0.39 | –1.47 |
| 4. Interrupts others | 1.09 | 1.08 | 0.47 | –1.15 |
| 5. Has a flaring temper and difficulties controlling himself/herself | 1.19 | 1.03 | 0.27 | –1.16 |
| 6. Throws things, when he/she loses his/her temper | 0.49 | 0.83 | 1.62 | 1.61 |
| 7. Does not follow rules of adults | 0.94 | 1.03 | 0.66 | –0.88 |
| 8. Frequently argues with adults | 0.84 | 0.92 | 0.67 | –0.74 |
| 9. Gets into physical fights with family/friends | 0.53 | 0.89 | 1.51 | 1.10 |
| 10. Gets into trouble for his/her rule breaking or antisocial behavior | **0.16** | 0.53 | **3.63** | **13.48** |
| 11. Spends time with other children/youth, who do not follow rules or are antisocial | **0.23** | 0.54 | **2.52** | 6.27 |
| 12. Lies to get something or gain advantages | 0.68 | 0.91 | 1.08 | –0.05 |
| 13. Annoy others on purpose | 0.52 | 0.82 | 1.48 | 1.22 |
| 14. Threatens or bullies others | **0.16** | 0.53 | **3.73** | **14.33** |
| Internalizing |  |  |  |  |
| 15. Finds it hard to experience joy or have fun | 0.59 | 0.94 | 1.33 | 0.39 |
| 16. Quickly starts to cry | 0.78 | 0.89 | 0.82 | –0.35 |
| 17. Appears unhappy or sad | 0.98 | 0.96 | 0.48 | –0.98 |
| 18. Feels worthless and has little self-confidence | 1.29 | 0.92 | 0.24 | –0.79 |
| 19. Has little or no energy | 0.60 | 0.94 | 1.30 | 0.32 |
| 20. Worries about a lot of things | 1.15 | 1.07 | 0.43 | –1.10 |
| 21. Is nervous/shy | 0.80 | 1.00 | 0.93 | –0.41 |
| 22. Feel tense | 0.98 | 1.13 | 0.64 | –1.10 |
| 23. Fears being laughed at | 0.85 | 1.01 | 0.92 | –0.36 |
| 24. Has difficulties falling asleep or sleeping through or has other sleeping problems | 1.15 | 1.15 | 0.41 | –1.33 |
| *Note.* The item order of the SFSS-I differs from that of the original SFSS instrument and items have been rearranged. Bold values indicate low means or values above the threshold. | | | | |

**Table S7**

*Interrater Reliability of the SFSS-I Scales between Two Additional Raters (*n *= 61)*

| Scale | ICC(2,1) | 95% CI | ICC(2,2) | 95% CI |
| --- | --- | --- | --- | --- |
| Internalizing | .96 | [.92, .98] | .98 | [.96, .99] |
| Externalizing | .93 | [.88, .96] | .97 | [.94, .98] |
| Total | .91 | [.83, .95] | .95 | [.91, .98] |
| *Note.* CI = confidence interval; ICC = interclass correlation coefficient; ICC(2,1) = two-way random-effects, absolute agreement model for single rater/measurements; ICC(2,2) = two-way random-effects, absolute agreement model based on a mean-rating of two additional raters (*k* = 2). | | | | |

**Table S8**

*Interrater Reliability on the Single Item Level (*n *= 61)*

| Item | ICC (1,1) | 95% CI | ICC (1,3) | 95% CI |
| --- | --- | --- | --- | --- |
| 1 | .63 | [.49, .74] | .83 | [.75, .90] |
| 2 | .88 | [.82, .92] | .96 | [.93, .97] |
| 3 | .91 | [.86, .94] | .97 | [.95, .98] |
| 4 | .81 | [.73, .87] | .93 | [.89, .95] |
| 5 | .65 | [.52, .76] | .85 | [.77, .90] |
| 6 | .86 | [.79, .91] | .95 | [.92, .97] |
| 7 | .86 | [.79, .91] | .95 | [.92, .97] |
| 8 | .75 | [.65, .83] | .90 | [.85, .94] |
| 9 | .88 | [.82, .92] | .96 | [.93, .97] |
| 10 | .78 | [.69, .85] | .92 | [.87, .95] |
| 11 | .53 | [.38, .66] | .77 | [.65, .85] |
| 12 | .83 | [.75, .89] | .93 | [.90, .96] |
| 13 | .87 | [.81, .92] | .95 | [.93, .97] |
| 14 | .67 | [.54, .77] | .86 | [.78, .91] |
| 15 | .66 | [.54, .77] | .86 | [.78, .91] |
| 16 | .89 | [.84, .93] | .96 | [.94, .98] |
| 17 | .89 | [.84, .93] | .96 | [.94, .98] |
| 18 | .75 | [.64, .83] | .90 | [.84, .94] |
| 19 | .57 | [.43, .70] | .80 | [.69, .87] |
| 20 | .91 | [.87, .94] | .97 | [.95, .98] |
| 21 | .85 | [.78, .90] | .94 | [.91, .96] |
| 22 | .82 | [.74, .88] | .93 | [.89, .96] |
| 23 | .91 | [.85, .94] | .97 | [.95, .98] |
| 24 | .91 | [.86, .94] | .97 | [.95, .98] |
| *Note.* ICC (1,1) = one-way random-effects, absolute agreement model for single rater/measurements; CI = confidence interval; ICC (1,3) = one-way random-effects, absolute agreement model based on a mean-rating of one interviewer and two additional raters (*k* = 3). | | | | |

**Table S9**

*Sensitivity and Specificity for all Potential Cut-Off Scores of the SFSS-I Internalizing and Externalizing Scales as well as Youden Index values (*N *= 358)*

| Cut-off points | Sensitivity | 95% CI | Specificity | 95% CI | Youden Index |
| --- | --- | --- | --- | --- | --- |
| SFSS-I Internalizing | | | | | |
| –1 | 1 | [1, 1] | 0 | [0, 0] | 0 |
| 0.5 | .994 | [.98, 1] | .047 | [.02, .08] | .041 |
| 1.5 | .982 | [.96, 1] | .126 | [.08, .17] | .108 |
| 2.5 | .982 | [.96, 1] | .179 | [.13, .24] | .161 |
| 3.5 | .958 | [.93, .98] | .258 | [.19, .32] | .216 |
| 4.5 | .935 | [.90, .97] | .368 | [.30, .44] | .303 |
| 5.5 | .875 | [.82, .92] | .474 | [.41, .54] | .349 |
| 6.5 | .827 | [.77, .88] | .547 | [.48, .62] | .374 |
| **7.5** | **.792** | **[.73, .85]** | **.621** | **[.55, .69]** | **.413** |
| 8.5 | .708 | [.64, .77] | .684 | [.62, .75] | .392 |
| 9.5 | .619 | [.55, .69] | .747 | [.68, .81] | .366 |
| 10.5 | .565 | [.49, .64] | .811 | [.75, .86] | .376 |
| 11.5 | .476 | [.40, .55] | .842 | [.79, .89] | .318 |
| 12.5 | .393 | [.32, .46] | .858 | [.81, .91] | .251 |
| 13.5 | .333 | [.26, .40] | .879 | [.83, .92] | .212 |
| 14.5 | .268 | [.20, .33] | .895 | [.85, .94] | .163 |
| 15.5 | .250 | [.18, .32] | .926 | [.89, .96] | .176 |
| 16.5 | .220 | [.16, .29] | .942 | [.91, .97] | .162 |
| 17.5 | .173 | [.12, .23] | .958 | [.93, .98] | .131 |
| 18.5 | .119 | [.07, .17] | .974 | [.95, .99] | .093 |
| 19.5 | .101 | [.06, .15] | .979 | [.96, .99] | .080 |
| 20.5 | .054 | [.02, .09] | .984 | [.96, 1] | .038 |
| 21.5 | .030 | [.01, .06] | .989 | [.97, 1] | .019 |
| 22.5 | .024 | [.01, .05] | .989 | [.97,1] | .013 |
| 23.5 | .018 | [0, .04] | .995 | [.98, 1] | .013 |
| 25.5 | .006 | [0, .02] | 1 | [1, 1] | .006 |
| 28 | 0 | [0, 0] | 1 | [1, 1] | 0 |
| SFSS-I Externalizing | | | | | |
| ­–1 | 1 | [1, 1] | 0 | [0, 0] | 0 |
| 0.5 | 1 | [1, 1] | .107 | [.07, .15] | .107 |
| 1.5 | .993 | [.98, 1] | .205 | [.15, .26] | .198 |
| 2.5 | .986 | [.97, 1] | .302 | [.24, .36] | .288 |
| 3.5 | .965 | [.93, .99] | .363 | [.30, .42] | .328 |
| 4.5 | .937 | [.90, .97] | .442 | [.38, .51] | .379 |
| 5.5 | .916 | [.87, .96] | .540 | [.47, .60] | .456 |
| 6.5 | .888 | [.83, .94] | .614 | [.55, .67] | .502 |
| 7.5 | .860 | [.80, .92] | .642 | [.58, .70] | .502 |
| 8.5 | .832 | [.77, .89] | .693 | [.63, .75] | .525 |
| 9.5 | .790 | [.72, .85] | .744 | [.68, .80] | .534 |
| **10.5** | **.769** | **[.70, .83]** | **.777** | **[.72, .83]** | **.546** |
| 11.5 | .706 | [.63, .78] | .814 | [.76, .87] | .520 |
| 12.5 | .678 | [.60, .75] | .851 | [.80, .90] | .529 |
| 13.5 | .615 | [.54, .69] | .874 | [.83, .92] | .489 |
| 14.5 | .573 | [.49, .66] | .888 | [.84, .93] | .461 |
| 15.5 | .524 | [.44, .61] | .916 | [.87, .95] | .440 |
| 16.5 | .455 | [.38, .54] | .926 | [.89, .96] | .381 |
| 17.5 | .406 | [.33, .49] | .940 | [.91, .97] | .346 |
| 18.5 | .329 | [.25, .41] | .940 | [.91, .97] | .269 |
| 19.5 | .308 | [.23, .38] | .958 | [.93, .98] | .266 |
| 20.5 | .273 | [.20, .34] | .963 | [.93, .99] | .236 |
| 21.5 | .238 | [.17, .31] | .967 | [.94, .99] | .205 |
| 22.5 | .210 | [.14, .28] | .967 | [.94, .99] | .177 |
| 23.5 | .175 | [.11, .24] | .986 | [.97, 1] | .161 |
| 24.5 | .154 | [.10, .22] | .991 | [.98, 1] | .145 |
| 25.5 | .133 | [.08, 19] | .991 | [.98, 1] | .124 |
| 26.5 | .105 | [.06, .15] | .995 | [.99, 1] | .100 |
| 27.5 | .063 | [.03, .10] | .995 | [.99, 1] | .058 |
| 28.5 | .056 | [.02, .09] | 1 | [1, 1] | .056 |
| 30 | .042 | [.01, .07] | 1 | [1, 1] | .042 |
| 31.5 | .035 | [.01, .06] | 1 | [1, 1] | .035 |
| 32.5 | .028 | [.01, . 06] | 1 | [1, 1] | .028 |
| 34.5 | .014 | [0, .03] | 1 | [1, 1] | .014 |
| 37 | .007 | [0, .02] | 1 | [1, 1] | .007 |
| 39 | 0 | [0, 0] | 1 | [1, 1] | 0 |
| *Note.* Bold values indicate the optimal cut-off points inclusive of sensitivity, specificity, 95% CI, and Youden Index. | | | | | |

**Table S10**

*Relationship between SFSS-I Cut-Off Score-Based Categorization (Internalizing Scale) and Psychotherapist-Assigned Diagnosis*

| Internalizing diagnosis | Cut-off categorization | | Total |
| --- | --- | --- | --- |
|  | No | Yes |  |
| No | 118 (TN) | 72 (FP) | 190 (D-) |
| Yes | 35 (FN) | 133 (TP) | 168 (D+) |
| Total | 153 (SFSS-I-) | 205 (SFSS-I+) | 358 (N) |

*Note.* TN = true negative; FN = false negative; FP = false positive; TP = true positive; D- = the total number of subjects who were not assigned with an internalizing diagnosis by a psychotherapist; D+ = the total number of subjects who were assigned with an internalizing diagnosis by a psychotherapist; SFSS-I- = total number of subjects with an internalizing subscale score on the SFSS-I < 7.5; SFSS-I+ = total number of subjects with an internalizing subscale score on the SFSS-I ≥ 7.5.

**Table S11**

*Relationship between SFSS-I Cut-Off Score-Based Categorization (Externalizing Scale) and Psychotherapist-Assigned Diagnosis*

| Externalizing diagnosis | Cut-off categorization | | Total |
| --- | --- | --- | --- |
|  | No | Yes |  |
| No | 167 (TN) | 48 (FP) | 215 (D-) |
| Yes | 33 (FN) | 110 (TP) | 143 (D+) |
| Total | 200 (SFSS-I-) | 158 (SFSS-I+) | 358 (N) |

*Note.* TN = true negative; FN = false negative; FP = false positive; TP = true positive; D- = the total number of subjects who were not assigned with an externalizing diagnosis by a psychotherapist; D+ = the total number of subjects who were assigned with an externalizing diagnosis by a psychotherapist; SFSS-I- = total number of subjects with an externalizing subscale score on the SFSS-I < 10.5; SFSS-I+ = total number of subjects with an externalizing subscale score on the SFSS-I ≥ 10.5.

**Table S12**

*Clinical Utility of SFSS-I Cut-Off Score Categorization (Internalizing Scale) in Relation to Psychotherapist-Assigned Diagnosis (*N *= 358)*

| Measures (metrics) | Formula | Results |
| --- | --- | --- |
| Sensitivity | TP/D+ | .79 |
| Specificity | TN/D- | .62 |
| Positive likelihood ratio | Sensitivity/(1-Specificity) | 2.08 |
| Negative likelihood ratio | (1-Sensitivity)/Specificity | .34 |
| Positive predictive value | TP/SFSS-I+ | .65 |
| Negative predictive value | TN/SFSS-I- | .77 |
| Accuracy | (TP+TN)/N | .70 |

*Note.* TN = true negative; TP = true positive; D- = the total number of subjects who were not assigned with an internalizing diagnosis by a psychotherapist; D+ = the total number of subjects who were assigned with an internalizing diagnosis by a psychotherapist; SFSS-I- = total number of subjects with an internalizing subscale score on the SFSS-I < 7.5; SFSS-I+ = total number of subjects with an internalizing subscale score on the SFSS-I ≥ 7.5.

**Table S13**

*Clinical Utility of SFSS-I Cut-Off Score Categorization (Externalizing Scale) in Relation to Psychotherapist-Assigned Diagnosis (*N *= 358)*

| Measures (metrics) | Formula | Results |
| --- | --- | --- |
| Sensitivity | TP/D+ | .77 |
| Specificity | TN/D- | .78 |
| Positive likelihood ratio | Sensitivity/(1-Specificity) | 3.50 |
| Negative likelihood ratio | (1-Sensitivity)/Specificity | .29 |
| Positive predictive value | TP/SFSS-I+ | .70 |
| Negative predictive value | TN/SFSS-I- | .85 |
| Accuracy | (TP+TN)/N | .77 |

*Note.* TN = true negative; TP = true positive; D- = the total number of subjects who were not assigned with an externalizing diagnosis by a psychotherapist; D+ = the total number of subjects who were assigned with an externalizing diagnosis by a psychotherapist; SFSS-I- = total number of subjects with an externalizing subscale score on the SFSS-I < 10.5; SFSS-I+ = total number of subjects with an externalizing subscale score on the SFSS-I ≥ 10.5.

**Table S14**

*Pre- to Post-Test Probabilities for the Presence and Absence of Internalizing and Externalizing Disorders Using the Proposed Cut-Off Scores (*N *= 358)*

|  | Cut-off score | Pre-test probability (%) | Post-test probability (%) | Change (absolute percentage points) |
| --- | --- | --- | --- | --- |
| Presence of an internalizing disorder | ≥ 7.5 | 46.9 | 64.8 | +17.9 |
| Absence of an internalizing disorder | < 7.5 | 46.9 | 23.1 | -23.8 |
| Presence of an externalizing disorder | ≥ 10.5 | 39.9 | 69.5 | +29.6 |
| Absence of an externalizing disorder | < 10.5 | 39.9 | 16.3 | -23.6 |
